# Effect of sivelestat sodium in patients with acute lung injury or acute respiratory distress syndrome: a meta-analysis of randomized controlled trials

**DOI:** 10.1186/s12890-017-0498-z

**Published:** 2017-11-21

**Authors:** Shenglan Pu, Daoxin Wang, Daishun Liu, Yan Zhao, Di Qi, Jing He, Guoqi Zhou

**Affiliations:** 1 Department of Respiratory Medicine, Second Affiliated Hospital of Chongqing Medical University, Chongqing, 400010 China; 2 Department of Respiratory and Critical Care Medicine, The First People’s Hospital of Zunyi, Zunyi, China

**Keywords:** Sivelestat sodium, Patients, Acute lung injury, Acute respiratory distress syndrome

## Abstract

**Background:**

Sivelestat is widely used in treating acute lung injury (ALI)/acute respiratory distress syndrome (ARDS), although the clinical efficacy of sivelestat remains controversial. This study aimed to evaluate the impact of sivelestat in patients with ALI/ARDS.

**Methods:**

Electronic databases, PubMed, Embase, and the Cochrane Library, were searched to identify trials through April 2017. Randomized controlled trials (RCTs) were included irrespective of blinding or language that compared patients with and without sivelestat therapy in ALI/ARDS. A random-effects model was used to process the data, and the relative risk (RR) and standard mean difference (SMD) with corresponding 95% confidence intervals (CIs) were used to evaluate the effect of sivelestat.

**Results:**

Six RCTs reporting data on 804 patients with ALI/ARDS were included. Overall, no significant difference was found between sivelestat and control for the risk of 28–30 days mortality (RR: 0.94; 95% CI: 0.71–1.23; *P* = 0.718). Sivelestat therapy had no significant effect on ventilation days (SMD: 0.05; 95% CI: −0.27 to 0.38; *P* = 0.748), arterial oxygen partial pressure (PaO2)/fractional inspired oxygen (FiO2) level (SMD: 0.48; 95% CI: −0.45 to 1.41; *P* = 0.315), and intensive care unit (ICU) stays (SMD: −9.87; 95% CI: −24.30 to 4.56; *P* = 0.180). The results of sensitivity analysis indicated that sivelestat therapy might affect the PaO_2_/FiO_2_ level in patients with ALI/ARDS (SMD: 0.87; 95% CI: 0.39 to 1.35; *P* < 0.001).

**Conclusions:**

Sivelestat therapy might increase the PaO_2_/FiO_2_ level, while it had little or no effect on 28–30 days mortality, ventilation days, and ICU stays. These findings need to be verified in large-scale trials.

**Electronic supplementary material:**

The online version of this article (10.1186/s12890-017-0498-z) contains supplementary material, which is available to authorized users.

## Background

Acute lung injury (ALI) or acute respiratory distress syndrome (ARDS) is characterized by abnormal pulmonary physiology and gas exchange properties [1, 2], which are common complications in various diseases, and is related to higher morbidity and mortality [3, 4]. Generally, the process of gas exchange is completed by mechanical ventilation. However, mechanical ventilation does not significantly reduce the mortality caused by ALI/ARDS. Rather, the lung injury could aggravated by ventilator due to surfactant deficiency and dysfunction, which associated with the exacerbation of atelectasis, increased formation of oedema, and impairment of local host defence [4–7]. Until now, the effect of most-employed treatment strategies, including high dose of steroids, aspirin, and ulinastain, in patients with ALI/ARDS remains limited [8].

Sivelestat is a neutrophil elastase inhibitor, which induces competitive inhibition of neutrophils, inhibition of neutrophil activation, and reduction of inflammation in the lungs [9, 10]. Currently, the use of sivelestatis already approved in Japan [11, 12]. However, the effectiveness of sivelestat in clinical needs is yet to be interpreted. Several randomized controlled trials (RCTs) have indicated that sivelestat therapy can improve ventilation days and arterial oxygen partial pressure (PaO2)/fractional inspired oxygen (FiO2), while the efficacy of sivelestat therapy on other outcomes in patients with ALI/ARDS remains controversial. Therefore, a systematic review and meta-analysis of available RCTs were conducted to evaluate the treatment effect of sivelestat.

## Methods

### Data sources, search strategy, and selection criteria

This review was conducted and reported according to the Preferred Reporting Items for Systematic Reviews and Meta-analysis Statement issued in 2009 (Additional file 1: Checklist S1) [13].

A systematic review and meta-analysis of RCTs published through April 2017 were conducted to identify trials of sivelestat for patients with ALI/ARDS. Electronic databases PubMed, Embase, and the Cochrane Library were searched using the following key words: (“sivelestat” OR “elaspol”) AND (“ARDS” OR “adult respiratory distress syndrome” OR “acute respiratory distress syndrome” OR “noncardiogenic pulmonary edema” OR “respiratory insufficiency” OR “systemic inflammatory response syndrome” OR “shock lung” OR “respiratory failure” OR “lung injury*” OR “septic shock” OR “sepsis”). Manual searches of the reference lists were also conducted from all relevant original and review articles to identify additional eligible studies. No language restriction was applied. Unpublished trials were excluded. The medical subject heading, methods, patient disease status, study design, intervention, and outcome variables were used to identify relevant studies.

The literature search was independently performed by two authors using a standardized approach. Any inconsistencies were settled by a group discussion until a consensus was reached. The included studies met the following criteria. (1) RCTs, (2) patients confirmed with ALI/ARDS, (3) patients received sivelestat, and (4) data included 28–30 days mortality, improved ventilation days, PaO_2_/FiO_2_ level, and intensive care unit (ICU) stays. All retrospective clinical studies that could affect the treatment effects due to various confounding biases were excluded.

The ethical approval and written consent are not necessary for the meta-analysis, because the data of meta-analysis is collected from published literature.

### Data collection and quality assessment

The data collected included the first author’s name, publication year, country, sample size, mean age, percentage of male, disease status, intervention, baseline PaO_2_/FiO_2_ ratio, baseline acute physiology and chronic health evaluation (APACHE II) score, reported endpoints, and study design variables. The authors independently scanned the titles and abstracts of the studies for eligibility and relevance. Potentially relevant articles were retrieved and reviewed for selection based on the inclusion and exclusion criteria. Any discrepancies were resolved by discussion. Further, the Jadad scale was employed to evaluate the methodological quality, based on randomization, concealment of treatment allocation, blinding, completeness of follow-up, and use of intention-to-treat analysis [14].

### Statistical analysis

Relative risks (RRs) and standard mean differences (SMDs) with 95% confidence intervals (CIs) were calculated using outcomes extracted from each study before data pooling. The random-effects model was used to calculate pooled RRs with 95% CI to estimate the effect of sivelestat on the risk of 28–30 days mortality, and SMDs were employed to estimate the efficacy of sivelestat therapy on the ventilation days, PaO_2_/FiO_2_ level, and ICU stays [15, 16]. Heterogeneity among trials was investigated using the Q statistic, and *P* values <0.10 were indicative of significant heterogeneity [17, 18]. Sensitivity analyses were conducted for ventilation days and PaO_2_/FiO_2_ level by removing each individual study from the meta-analysis [19]. The subgroup analysis was also performed for 28–30 days mortality based on publication year, mean age, percentage of male, disease status, baseline PaO_2_/FiO_2_ ratio, and Jadad score. The visual inspection of funnel plots for 28–30 days mortality was conducted. The Egger [20] and Begg [21] tests were also used to statistically assess the publication bias for 28–30 days mortality. All reported *P* values were two sided, and *P* values <0.05 were considered as statistically significant. Statistical analyses were performed using the STATA software (version 10.0; Stata Corporation, TX, USA).

## Results

**Fig. 1:**
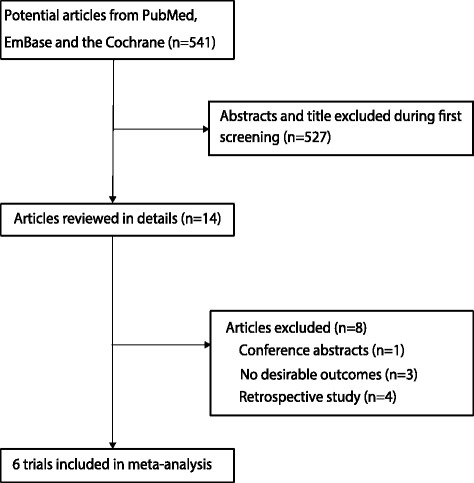
Flow diagram of the literature search and trials selection process

The results of the study-selection process are shown in Fig. 1. A total of 541 potentially relevant articles were identified after systematically searching electronic databases, professional journals, and other sources. After reviewing the titles or abstracts, 527 were excluded as they did not meet the inclusion criteria, leaving 14 articles for further full-text reviews. Six RCTs were finally identified and included for the analysis of treatment effect of sivelestat in patients with ALI/ARDS [22–27], and the rest were excluded for the following reasons: conference abstracts without full text, retrospective study, and no desirable outcomes. A manual search of the reference lists of these trials did not yield any new eligible studies. The general characteristics of the included studies are presented in Table 1.

**Table 1:** Baseline characteristic of studies included in the systematic review and meta-analysis

Study	Publication year	Country	Sample size	Mean age	Percentage male (%)	Disease status	Intervention	Baseline PaO_2_/FiO_2_ ratio	Baseline APACHE II score	Jadad scale
Endo [22]	2006	Japan	26	NA	NA	ALI	0.2 mg/kg/h for 14 days	NA	NA	3
Sato [23]	2008	Japan	24	69.0	75.0	ALI/ARDS	0.2 mg/kg/h for 14 days	196.5	NA	2
Morimoto [24]	2011	Japan	22	73.1	63.6	ALI	0.2 mg/kg/h huntil weaning from mechanical ventilation	<150.0	NA	3
Kadoi [25]	2004	Japan	24	64.0	75.0	ARDS	0.2 mg/kg/h for 14 days	148.5	20.1	2
Zeiher [26]	2004	Multiple countries	487	56.0	59.3	ALI	0.16 mg/kg/h for 14 days	148.7	20.8	3
Tamakuma [27]	2004	Japan	221	57.8	76.0	ALI	0.2 mg/kg/h for 14 days	199.0	NA	3

*ALI* Acute lung injury, *APACHE II* acute physiology and chronic health evaluation, *ARDS* acute respiratory distress syndrome, *FiO*_*2*_ fractional inspired oxygen, *PaO*_*2*_ arterial oxygen partial pressure

Six RCTs involving a total of 804 patients with ALI/ARDS were included. The mean age of the patients was 56.0–73.1 years. Each trial included 22–487 individuals. Further, the percentage of included males ranged from 59.3%–76.0%. Five trials were conducted in Japan [22–25, 27], and the remaining one trial in multiple countries [26]. Four of the included trials reported patients with ALI [22, 24, 26, 27], one trial included patients with ARDS [25], and the remaining one trial included patients with both ALI and ARDS [23]. Moreover, five trials included patients who received 0.2 mg/kg/h sivelestat [22–25, 27], and one trial included those who received 0.16 mg/kg/h sivelestat [26]. The study quality was assessed using the Jadad score and is presented in Table 1. Overall, four trials had a score of 3 [22, 24, 26, 27], and the remaining two had a score of 2 [23, 25].

**Fig. 2:**
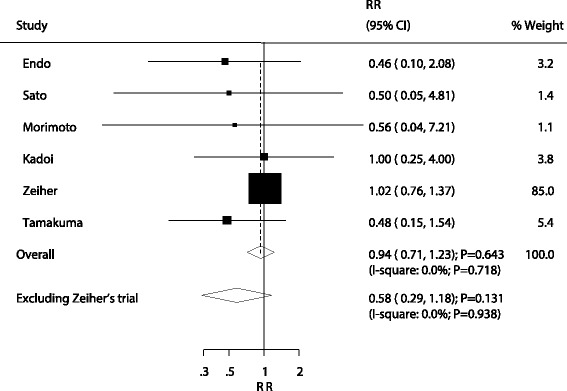
Effect of sivelestat on the risk of 28–30 days mortality

All included trials reported the effect of sivelestat on the risk of 28–30 days mortality. The summary results indicated no significant difference between sivelestat and control for the risk of 28–30 days mortality (RR: 0.94; 95% CI: 0.71–1.23; *P* = 0.643; Fig. 2), and without evidence of heterogeneity. The sensitivity analysis found that the risk of 28–30 days mortality was reduced by 42%, but was not statistically significant when excluding the study by Zeiher et al. (RR: 0.58; 95% CI: 0.29–1.18; *P* = 0.131; Fig. 2). This trial specifically included a higher incidence of mortality within 28–30 days and included patients who received low-dose sivelestat therapy.

**Fig. 3:**
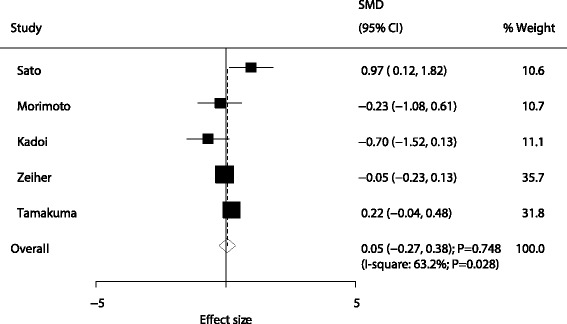
Effect of sivelestat therapy on ventilation days

A total of five trials reported the effect of sivelestat therapy on ventilation days in patients with ALI/ARDS. No significant difference was found between sivelestat and control for ventilation days (SMD: 0.05; 95% CI: −0.27 to 0.38; *P* = 0.748; Fig. 3). Although substantial heterogeneity was observed in the magnitude of the effect across the studies (*P* = 0.028), the conclusion was not affected by the exclusion of any specific study after the sequential exclusion of each study from all of the pooled analyses (Table 2).

**Table 2:** Sensitivity analysis for ventilation days and PaO_2_/FiO_2_

Outcomes	Excluding study	SMD (95% CI)	*P* value	Heterogeneity (%)	*P* value for heterogeneity
Ventilation days	Sato	−0.02 (−0.29 to 0.25)	0.865	50.7	0.107
	Morimoto	0.09 (−0.27 to 0.45)	0.633	71.4	0.015
	Kadoi	0.14 (−0.17 to 0.45)	0.388	61.3	0.052
	Zeiher	0.08 (−0.48 to 0.64)	0.766	65.4	0.034
	Tamakuma	−0.02 (−0.54 to 0.50)	0.950	62.9	0.044
PaO_2_/FiO_2_	Morimoto	0.37 (−0.73 to 1.47)	0.507	97.4	<0.001
	Kadoi	0.53 (−0.58 to 1.64)	0.350	97.4	<0.001
	Zeiher	0.18 (−0.53 to 0.89)	0.623	73.2	0.024
	Tamakuma	0.87 (0.39 to 1.35)	<0.001	49.1	0.140

*CI* confidence interval, *FiO*_*2*_ fractional inspired oxygen, *PaO*_*2*_ arterial oxygen partial pressure, *SMD* standard mean difference, *RR* relative risk

**Fig. 4:**
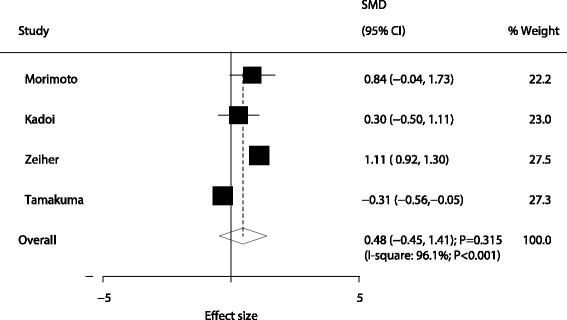
Effect of sivelestat therapy on PaO_2_/FiO_2_

**Fig. 5:**
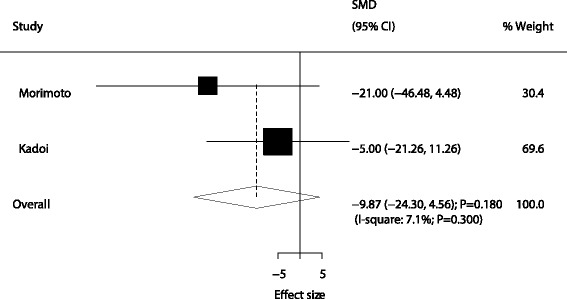
Effect of sivelestat therapy on ICU stays

A total of four trials reported the effect of sivelestat therapy on PaO_2_/FiO_2_ in patients with ALI/ARDS. It was noted that the PaO_2_/FiO_2_ level in patients with ALI/ARDS who received sivelestat therapy had increased by 0.48, although it was not statistically significant (SMD: 0.48; 95% CI: −0.45 to 1.41; *P* = 0.315; Fig. 4), and the potential evidence of significant heterogeneity was detected (*P* < 0.001). According to the sensitivity analysis, the study by Tamakuma et al. was excluded because it specifically included patients with higher baseline PaO_2_/FiO_2_ level and might affect the treatment effect of sivelestat therapy. After this exclusion, it was concluded that sivelestat therapy significantly increased the level of PaO_2_/FiO_2_ in patients with ALI/ARDS (SMD: 0.87; 95% CI: 0.39 to 1.35; *P* < 0.001; Table 2). Moreover, sivelestat therapy had little or no effect on ICU stays in patients with ALI/ARDS (SMD: −9.87; 95% CI: −24.30 to 4.56; *P* = 0.180; Fig. 5) (Table 3).

**Table 3:** Subgroup analyses for 28–30 days mortality excluding the study conducted by Zeiher et al.

Group	RR (95% CI)	*P* value	Heterogeneity (%)	*P* value for heterogeneity	*P* value for interaction test
Publication year					
2005 or after	0.49 (0.16–1.52)	0.215	0.0	0.992	0.696
Before 2005	0.65 (0.27–1.59)	0.344	0.0	0.427	
Mean age (years)					
≥ 65.0	0.53 (0.09–2.92)	0.462	0.0	0.949	0.897
< 65.0	0.60 (0.28–1.28)	0.186	0.0	0.677	
Percentage male (%)					
≥ 70.0	0.63 (0.27–1.44)	0.272	0.0	0.713	0.741
< 70.0	0.48 (0.13–1.79)	0.277	0.0	0.898	
Disease status					
ALI	0.48 (0.20–1.15)	0.100	0.0	0.992	0.468
ALI/ARDS or ARDS	0.83 (0.25–2.71)	0.757	0.0	0.611	
Baseline PaO_2_/FiO_2_ ratio					
≥ 150	0.48 (0.17–1.37)	0.170	0.0	0.975	0.634
< 150	0.68 (0.26–1.77)	0.430	0.0	0.751	
Jadad score					
3	0.48 (0.20–1.15)	0.100	0.0	0.992	0.468
2	0.83 (0.25–2.71)	0.757	0.0	0.611	

*ALI* Acute lung injury, *ARDS* acute respiratory distress syndrome, *CI* confidence interval, *FiO*_*2*_ fractional inspired oxygen, *PaO*_*2*_ arterial oxygen partial pressure, *RR* relative risk

**Fig. 6:**
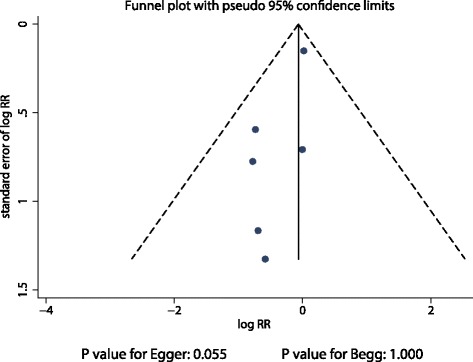
Funnel plot for 28–30 days mortality

The publication bias was assessed using the funnel plot for 28–30 days mortality (Fig. 6). Although the Begg test showed no evidence of publication bias for 28–30 days mortality, the Egger test showed potential evidence of publication bias. However, the results were not influenced after adjustment for publication bias using the trim-and-fill method [28].

## Discussion

The objective of the present meta-analysis was to evaluate the effect of sivelestat therapy in patients with ALI/ARDS. Six trials including 804 patients with ALI/ARDS were included. The summary results showed that sivelestat therapy had little or no significant effect on 28–30 days mortality, ventilation days, PaO_2_/FiO_2_ level, and ICU stays. The findings of the sensitivity analysis indicated that sivelestat therapy might play a beneficial effect on the level of PaO_2_/FiO_2_. These results might help better define the treatment effect of sivelestat therapy in patients with ALI/ARDS and help physicians to select appropriate treatment strategies.

A previous meta-analysis including eight trials suggested that sivelestat therapy was not associated with 28–30 days mortality and mechanical ventilation days, while it was associated with lower PaO_2_/FiO_2_ ratio in patients with ALI/ARDS [29]. The study did not recommend its routine use in patients with ALI/ARDS. The effect of sivelestat therapy on ICU stays was not conducted, and the treatment effects according to different baseline characteristics were not performed. Therefore, the present study conducted a comprehensive systematic review and meta-analysis to evaluate the effect of sivelestat therapy in patients with ALI/ARDS.

The findings of the present study suggested that sivelestat therapy had no significant effect on 28–30 days mortality. All included trials reported that sivelestat therapy did not affect the risk of mortality within 28–30 days. However, nearly all trials reported that the incidence of 28–30 days mortality reduced but was not statistically significant. Moreover, Zeiher et al. found that sivelestat therapy was associated with a nonsignificant increase in the risk of 28 days mortality by 2% [26]. The possible reason could be the efficacy of sivelestat on 28 days mortality which might be affected by specific clinical conditions [30]. Meanwhile, sivelestat has maximum efficacy in patients with mild to moderate ARDS [31]. Further, the treatment effects of sivelestat were correlated with age, disease status, haemodialysis, and methylprednisoline use [32]. Furthermore, subgroup analyses for 28 days mortality, excluding the study conducted by Zeiher et al., were performed [26]. The findings of subgroup analysis were consistent with the overall analysis.

No significant difference was found between sivelestat therapy and control for ventilation days. Mostly included trials indicated that sivelestat therapy had no significant effect on ventilation days, while the trial conducted by Sato et al. reported inconsistent results [23]. This study specifically included patients with both ALI and ARDS, which might affect ventilation days. Further, the Sato’s study might include more severe ALI/ARDS patients, which was associated with lower respiratory function so that sivelestat became less effective [33]. Finally, sivelestat therapy did not affect the PaO_2_/FiO_2_ level, and ICU stays in ALI/ARDS patients. However, these conclusions may be variable since a smaller number of trials were included. Therefore, the present study gave a relative result and provided a synthetic and comprehensive review.

The strengths of this meta-analysis were as follows: (1) the large sample size allowed the quantitative assessment of the efficacy of sivelestat, and thus these findings were potentially more robust than any individual study. Second, the results of ICU stays were summarized, as the previous meta-analysis was not conducted. Third, the treatment effect of sivelestat in patients with ALI/ARDS according to different baseline characteristics was conducted, which provided any potential effect of sivelestat therapy in specific subpopulations.

The limitation of this study were as follows: (1) the number of included studies was smaller than expected, which always acquired broad CIs, that is, no statistically significant difference; (2) data on baseline APACHE score of the enrolled patients were available in two trials, which might affect the treatment effect of sivelestat in ALI/ARDS patients [25, 26]; (3) the information about ALI/ARDS classification were available in two trials [26, 27] and other trials could not provide diagnosis criteria of ALI/ARDS patients, which was correlated with the treatment effects of sivelestat; (4) mostly included trials were conducted in Japan, which might induce ethnic biases; (5) in a meta-analysis of published studies, publication bias is inevitable; and (6) the analysis used pooled data (individual data were not available), which prevented a detailed analysis to obtain more comprehensive results.

## Conclusion

The findings of this study suggested that sivelestat therapy might play an important role on the PaO_2_/FiO_2_ level, while it had no significant effect on 28–30 days mortality, ventilation days, and ICU stays. Future large-scale trials should focus on different disease status, patient characteristics, and trials from other countries to analyze any possible efficacy and safety of sivelestat therapy.

## Additional file

Additional file 1: Prisma 2009 Checklist. (DOC 72 kb)

## Abbreviations

ALI: Acute lung injury
ARDS: Acute respiratory distress syndrome
CIs: Confidence intervals
FiO2: Fractional inspired oxygen
ICU: Intensive care unit
PaO2: Arterial oxygen partial pressure
RCTs: Randomized controlled trials
RR: Relative risk
SMD: Standard mean difference

## References

[1] Lynch JE, Cheek JM, Chan EY, Zwischenberger JB (2006). Adjuncts to mechanical ventilation in ARDS. Semin Thorac Cardiovasc Surg.

[2] Ashbaugh DG, Bigelow DB, Petty TL, Levine BE (1967). Acute respiratory distress in adults. Lancet.

[3] Janz DR, Ware LB (2014). Approach to the patient with the acute respiratory distress syndrome. Clin Chest Med.

[4] Tu X, Wang X, Chen W. Nursing care of patients with acute respiratory distress syndrome accepting NO inhalation. Chin Nurs Res. 2008;

[5] Liao P, Whitehead T, Evans T, Griffiths M (2003). Ventilator-associated lung injury. Lancet.

[6] Chiumello D, Pristine G, Slutsky AS (1999). Mechanical ventilation affects local and systemic cytokines in an animal model of acute respiratory distress syndrome. Am J Respir Crit Care Med.

[7] Tremblay L, Valenza F, Ribeiro SP, Li J, Slutsky SA (1997). Injurious ventilatory strategies increase cytokines and c-fos m-RNA expression in an isolated rat lung model. J Clin Invest.

[8] Adhikari N, Burns KE, Meade MO (2004). Pharmacologic treatments for acute respiratory distress syndrome and acute lung injury: systematic review and meta-analysis. Treat Respir Med.

[9] Hagio T, Matsumoto S, Nakao S, Abiru T, Ohno H, Kawabata K (2004). Elastase inhibition reduced death associated with acid aspiration-induced lung injury in hamsters. Eur J Pharmacol.

[10] Chai JK, Cai JH, Deng HP, Zou XF, Liu W, Hu QG (2013). Role of neutrophil elastase in lung injury induced by burn-blast combined injury in rats. Burns.

[11] Hayakawa M, Katabami K, Wada T, Sugano M, Hoshino H, Sawamura A (2010). Sivelestat (selective neutrophil elastase inhibitor) improves the mortality rate of sepsis associated with both acute respiratory distress syndrome and disseminated intravascular coagulation patients. Shock.

[12] Okayama N, Kakihana Y, Setoguchi D, Imabayashi T, Omae T, Matsunaga A (2006). Clinical effects of a neutrophil elastase inhibitor, sivelestat, in patients with acute respiratory distress syndrome. J Anesth.

[13] Moher D, Liberati A, Tetzlaff J, Altman DG, Group TP (2010). Preferred reporting items for systematic reviews and meta-analyses: the PRISMA statement. Revista Española De Nutrición Humana Y Dietética.

[14] Jadad AR, Moore RA, Carroll D, Jenkinson C, Reynolds DJ, Gavaghan DJ (1996). Assessing the quality of reports of randomized clinical trials: is blinding necessary?. Control Clin Trials.

[15] Dersimonian R, Laird N (1986). Meta-analysis in clinical trials. Control Clin Trials.

[16] Ades AE, Lu G, Higgins JPT (2005). The interpretation of random-effects meta-analysis in decision models. Med Decis Mak.

[17] Deeks JJ, Higgins JPT, Altman DG, Higgins J, Green S (2008). Analysing Data and Undertaking Meta-Analyses. Cochrane handbook for systematic reviews of interventions 5.0.1.

[18] Higgins JP, Thompson SG, Deeks JJ, Altman DG (2003). Measuring inconsistency in meta-analyses. Br Med J.

[19] Tobias A (1999). Assessing the influence of a single study in meta-analysis. Stata Tech Bull.

[20] Egger M, Smith GD, Schneider M, Minder C (1997). Bias in meta-analysis detected by a simple, graphical test. BMJ.

[21] Begg CB, Mazumdar M (1994). Operating characteristics of a rank correlation test for publication bias. Biometrics.

[22] Endo S, Sato N, Yaegashi Y, Suzuki Y, Kojika M, Yamada Y (2006). Sivelestat sodium hydrate improves septic acute lung injury by reducing alveolar dysfunction. Res Commun Mol Pathol Pharmacol.

[23] Sato N, Imai S, Yaegashi Y (2003). The effect of use of Elaspol on acute lung injury (ALI) with sepsis. Prog Med.

[24] Morimoto K, Nishimura K, Miyasaka S, Maeta H, Taniguchi I (2011). The effect of sivelestat sodium hydrate on severe respiratory failure after thoracic aortic surgery with deep hypothermia. Ann Thorac Cardiovasc Surg.

[25] Kadoi Y, Hinohara H, Kunimoto F, Saito S, Goto F, Kosaka T (2004). Pilot study of the effects of ONO-5046 in patients with acute respiratory distress syndrome. Anesth Analg.

[26] Zeiher BG, Artigas A, Vincent JA, Jackson K, Thompson BT, Bernard G (2004). Neutrophil elastase inhibition in acute lung injury: results of the STRIVE study. Crit Care Med.

[27] Tamakuma S, Ogawa M, Aikawa N, Kubota T, Hirasawa H, Ishizaka A (2004). Relationship between neutrophil elastase and acute lung injury in humans. Pulm Pharmacol Ther.

[28] Duval S, Tweedie R (2000). A nonparametric “trim and fill” method of accounting for publication bias in meta-analysis. J Am Stat Assoc.

[29] Iwata K, Doi A, Ohji G, Oka H, Oba Y, Takimoto K (2010). Effect of neutrophil elastase inhibitor (sivelestat sodium) in the treatment of acute lung injury (ALI) and acute respiratory distress syndrome (ARDS): a systematic review and meta-analysis. Intern Med.

[30] Aikawa N, Kawasaki Y (2014). Clinical utility of the neutrophil elastase inhibitor sivelestat for the treatment of acute respiratory distress syndrome. Ther Clin Risk Manag.

[31] Tsushima K, Yokoyama T, Matsumura T, Koizumi T, Kubo K, Tatsumi K (2014). The potential efficacy of noninvasive ventilation with administration of a neutrophil elastase inhibitor for acute respiratory distress syndrome. J Crit Care.

[32] Kido T, Muramatsu K, Yatera K, Asakawa T, Otsubo H, Kubo T (2017). Efficacy of early sivelestat administration on acute lung injury and acute respiratory distress syndrome. Respirology.

[33] Ozawa T, Mihara K, Yasuno N (2016). Predictors of the therapeutic effect of sivelestat in patients with acute lung injuryassociated with systemic inflammatory response syndrome. J Pharm Health Care Sci.

